# Social Agonistic Distress in Male and Female Mice: Changes of Behavior and Brain Monoamine Functioning in Relation to Acute and Chronic Challenges

**DOI:** 10.1371/journal.pone.0060133

**Published:** 2013-04-02

**Authors:** Shlomit Jacobson-Pick, Marie-Claude Audet, Robyn Jane McQuaid, Rahul Kalvapalle, Hymie Anisman

**Affiliations:** Department of Neuroscience, Carleton University, Ottawa, Canada; Nathan Kline Institute for Psychiatric Research and New York School of Medicine, United States of America

## Abstract

Stressful events promote several neuroendocrine and neurotransmitter changes that might contribute to the provocation of psychological and physical pathologies. Perhaps, because of its apparent ecological validity and its simple application, there has been increasing use of social defeat (resident-intruder) paradigms as a stressor. The frequency of stress-related psychopathology is much greater in females than in males, but the typical resident-intruder paradigm is less useful in assessing stressor effects in females. An alternative, but infrequently used procedure in females involves exposing a mouse to a lactating dam, resulting in threatening gestures being expressed by the resident. In the present investigation we demonstrated the utility of this paradigm, showing that the standard resident-intruder paradigm in males and the modified version in females promoted elevated anxiety in a plus-maze test. The behavioral effects that reflected anxiety were more pronounced 2 weeks after the stressor treatment than they were 2 hr afterward, possibly reflecting the abatement of the stress-related of hyper-arousal. These treatments, like a stressor comprising physical restraint, increased plasma corticosterone and elicited variations of norepinephrine and serotonin levels and turnover within the prefrontal cortex, hippocampus and central amygdala. Moreover, the stressor effects were exaggerated among mice that had been exposed to a chronic or subchronic-intermittent regimen of unpredictable stressors. Indeed, some of the monoamine changes were more pronounced in females than in males, although it is less certain whether this represented compensatory changes to deal with chronic stressors that could result in excessive strain on biological systems (allostatic overload).

## Introduction

Stressful events give rise to several neurochemical changes that promote emotional and behavioral responses that might facilitate an organism’s ability to deal with the stressor or to blunt some of the adverse consequences that might otherwise occur. Along with other biological responses, stressors elicit an increase in the release of norepinephrine (NE) and serotonin (5-HT) in the prefrontal cortex, hippocampus and amygdala, brain regions that have been associated with anxiety and depression [1]. The adaptive value of these neurochemical changes notwithstanding, if the stressor is sufficiently intense and persistent, the strain on these biological processes may become excessive, leading to allostatic overload and the development of psychological and physical disturbances [2].

In humans, stressor-related psychopathologies, including depression and anxiety disorders, are more common in females than in males [3], [4]. However, based on studies in animals it was suggested that females are actually more biologically resilient to the adverse effects of stressors, and the greater propensity to psychopathology might be related to psychosocial factors, including those related to stressor appraisals and coping methods [5]. It is also possible that women generally encounter more stressors than do men. It has indeed been proposed that estradiol might contribute to the greater stress-resilience of females, as the greater resistance to behavioral impairments elicited by stressors among females is diminished in older animals in which estradiol levels have declined [6]. Moreover, females typically exhibit greater elevations of corticosterone in response to stressors, possibly owing to greater influences of serotonergic functioning in females than in males [7]. Indeed, sex differences in response to stressors were eliminated in rats in which the 5-HTT transporter was knocked out (SERT −/−) [8]. In addition to serotonergic processes, the greater variations of NE changes elicited by stressors in females might contribute to resilience to memory impairments that are more often engendered in males [9]. The suggestion here is that the greater neurochemical reactivity to stressors in females reflects adaptive changes to meet environmental demands, rather than reflecting greater vulnerability to pathogenic outcomes; however, it is equally possible that the greater neurobiological reactivity in females might also render them more prone to allostatic overload so that with sustained stressor experiences pathology could arise more readily. Of course, it is difficult to define when or at what point the response to a stressor is one that is advantageous, and when the response becomes one that favors adverse outcomes.

The effects of stressors on pathology might vary as a function the characteristics of the stressor. For instance, uncontrollable stressors are particularly apt to engender behavioral and neurochemical disturbances, relative to those elicited by controllable stressors [1], [10]. As well, if the same stressor occurs repeatedly an ‘adaptation’ seems to occur so that some of the neurochemical change ordinarily elicited by an acute stressor becomes progressively less pronounced, and behavioral disturbances might be less likely to occur. However, if the chronic stressor experience involves a series of different stressors and occurs on a relatively unpredictable basis, then the adaptation is less likely to develop, and, in fact, the neurochemical changes may become progressively greater and the behavioral disturbances more profound [11].

Likely owing to their apparent ethological validity and simplicity, social stressors (e.g., social disruption and social defeat) have increasingly been used to identify processes potentially related to psychopathology. For instance, social defeat may promote reduced glucocorticoid receptor sensitivity [12] and increased central monoamine activity that might contribute to anxiety [13], [14]. Social defeat may also reduce levels of brain-derived neurotrophic factor (BDNF) [15] and DeltaFosB [16] within mesolimbic brain regions, and may influence hippocampal microvasculature [17] and neurogenesis [18], [19], thereby promoting vulnerability to memory disturbances and depressive-like states.

Although social defeat might be useful in modeling anxiety, depression or PTSD in males [20]–[25], there has been limited information concerning the effects of social defeat in females. Whereas a male intruder represents a threat to the male resident, which thus prompts aggression, the male behavior directed towards a female intruder in this paradigm is not similarly motivated, and hence aggressive attacks are less common. An alternative strategy to assess social aggression in females is to have an intruder placed in the cage of a lactating dam [26]–[28], which elicits aggressive displays that threaten the intruder, although these displays typically are not accompanied by overt attacks.

The present investigation was conducted to assess the effects of an acute social disturbance (defeat in males and threat from a lactating dam in females) on the levels of brain norepinephrine and serotonin and on their metabolites, in male and female mice. To be sure, the relative aversiveness of these very different stressors in males and females could not be compared to one another, and hence it was also of interest to establish whether or not the social stressors would elicit effects reminiscent of those provoked by a common stressor (restraint) in males and females. In a second experiment we assessed whether the response to the social stressors in males and females would be modified when defeat followed a chronic or subchronic-intermittent unpredictable stressor regimen. Finally, a third study assessed the effects of these stressor treatments on anxiety reflected by behavior on an elevated plus maze test.

## General Methods

### Subjects

Male and female CD-1 mice, approximately 70 days of age, that had been bred at Carleton University from parent stock obtained from Charles River Canada (St. Constant, Quebec) were used. The mice were housed with their same-sex siblings in groups of 3 in standard (27×21×14 cm) polypropylene cages (Experiment 1). As mice in Experiments 2 and 3 were exposed to repeated stressor exposure, mice in these studies were housed individually to preclude increased fighting that might otherwise be engendered. Mice were maintained on a 12-h light-dark cycle (light phase: 0700–1900 h), with temperature (22°C) and humidity (63%) kept constant, and were provided with free access to food (Ralston Purina) and water. The studies met the guidelines set out by the Canadian Council on Animal Care and were approved by the Carleton University Animal Care Committee (P10-6).

### Stressor Procedures

#### Experiment 1: Restraint and social stressor effects

**Figure 1:**
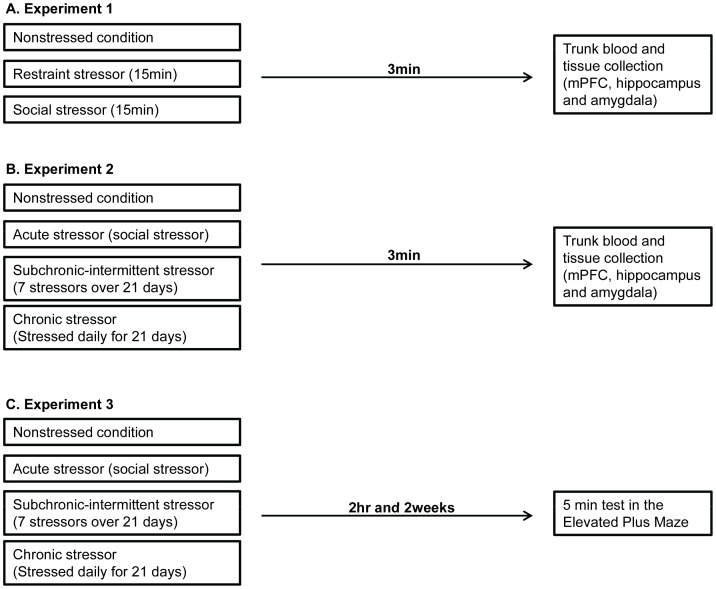
Experimental design of experiments 1, 2 and 3. In Experiment 1, mice were assigned to either nonstressed, a restraint stressor, or a social stressor conditions. 3 minutes following the stressor session, trunk blood was collected for corticosterone determination. Mice brains were rapidly removed and mPFC, hippocampus and amygdala were collected for subsequent High-performance liquid chromatography (HPLC) analyses (Fig. 1A). In Experiment 2, male and female mice were assigned to either a nonstressed, acute stressor, chronic stressor, or subchronic-intermittent stressor. Nonstressed mice left undisturbed over the course of the experiment, the acute stressor group were exposed to a single social stressor, chronic stressor group were stressed daily for 21 consecutive days and the subchronic- intermittent condition mice were stressed 7 times over 21 days period. Mice in the chronic stressor and subchronic- intermittent conditions were exposed to the following stressors: tight restraint, forced swim, wet bedding, tail pinch, soiled bedding, startle stimulus and on the last day mice undergone social stressor. Three minutes after the social stressor, mice were decapitated and blood and brain samples collected as described in Experiment 1 (Fig. 1B). Experiment 3, was conducted to assess the effects of the stressor conditions, precisely as described in Experiment 2, on anxiety-related behaviors measured on an elevated plus maze 2 hr and then again 2 weeks following exposure to the stressor conditions (Fig. 1C).

All procedures were conducted between 0830 and 1300 hours to minimize effects related to diurnal factors. The experimental design for each of the three experiments is provided in Figure 1. In Experiment 1, mice (N = 8 or 9/group) were assigned to either nonstress condition, an acute restraint stressor condition, or an acute social stressor. The restraint stressor comprised placing mice in a tightly fitting conically shaped plastic baggie with the end removed to allow the mouse’s snout to protrude. The bag was sealed by tape with the mouse’s tail protruding, thereby preventing it from turning. The baggy was placed on a flat surface for 15 min under room light conditions.

The social stressor among male mice comprised the resident-intruder paradigm in which an experimental mouse was placed in the home cage of a larger retired breeder for a 15 min period. In our studies social defeat is defined as the larger mouse visibly intimidating and threatening the intruder. In some cases attacks against the intruder occurred, but excessive aggression and injuries were limited by having an observer lightly shake or knock on the side of the cage if an attack was repeated. As in our previous studies, the social stressor among females comprised placing a naïve female in the cage of a lactating dam for 15 min (pups were removed immediately prior to the intruder being placed in the cage). Otherwise, the procedure in females was identical to that used in males.

After the stressor session mice were decapitated and trunk blood collected in tubes containing 10 mg EDTA, centrifuged, and the plasma stored at −80° for subsequent corticosterone determination. Brains were rapidly removed and placed on a stainless-steel brain matrix (2.5×3.75×.0 cm) that had been placed on a block of ice. The matrix had a series of slots spaced ∼500 µm apart that guided razor blades, thus providing coronal brain sections. Once slices were obtained, tissue was collected by micropunch from the mPFC, hippocampus and amygdala following the mouse atlas of Franklin and Paxinos [29]. Tissue samples were stored at 80°C for subsequent HPLC analyses.

#### Experiment 2: Influence of chronic stressors on subsequent corticosterone and brain monoamine responses elicited by the social stressor

Chronic social defeat is often used to model depression, although it has been shown that a single social defeat produces robust activation of the HPA-axis and induces variations of monoamine and GABA levels and turnover [13], [30]–[32]. We have also shown that a chronic variable stressor regimen, followed by an acute social stressor, elicits marked behavioral changes as well as variations of peripheral and central cytokine mRNA expression that might be relevant for depression [33]. Experiment 2 assessed whether or not the effects of the social defeat stressor would be moderated in mice that had previously been exposed to either a chronic or subchronic-intermittent variable, unpredictable stressor regimen. As depicted in Figure 1, male and female mice (N = 10/group) were assigned to either a nonstressed, acute stressor (single stressor session), chronic stressor, or subchronic-intermittent stressor condition. As the chronic stressor treatments could potentially elicit aggression amongst cage-mates, all mice were individually housed beginning 7 days prior to the experiment beginning despite the possibility that individual housing itself could elicit stressor-like effects.

Nonstressed mice were left in their cages, undisturbed over the course of the experiment, other than to have bedding changed (twice each week). Mice in the chronic stressor condition were exposed to a stressor on each of 21 days. In this regard, a chronic variable stressor procedure was used, but the nature of the stressor and their severities were more intense than that used in a chronic mild stress (CMS) model [34]–[35] as we found that such procedures provoked marked behavioral and biological disturbances [33]–[36]. Specifically, the stressors comprised 7 different challenges, one of which was applied on each day on the basis of a predetermined random sequence: Tight restraint - mice were restrained in tight fitting baggy for 15 minutes [31], [37], [38]; forced swim - placement in a bucket containing water (temperature 20–21°) for 15 minutes [37], [39]; wet bedding – mice were placed (1 hr) in a cage with water-soaked bedding [40]; tail pinch - a paper clip was enclosed around their tails for 5 minutes (a square piece of gauze was wrapped around mice’ tails before the clip was placed to minimize discomfort) [41]; soiled bedding – the bedding in mouse cage was replaced by dirty (used) bedding (24 hours) [42] **;** Startle stimulus - mice were placed in cylinder-shaped Plexiglas chamber for 15 minutes, during which they were exposed to ten 100 db stimuli (350 ms each) startle stimuli at random intervals [37]. Finally, on the 22^nd^ day, mice were challenged using the social stressor that had been used for the acutely stressed male and female mice [31]. Three minutes afterward, mice were decapitated and blood and brain samples collected as described in Experiment 1.

The subchronic-intermittent stressor procedure was also applied over a 21 day period, just as the chronic stressor had been. However, over the course of this period mice were only stressed on 7 days (i.e., on average of every 3^rd^ day on a random basis, following the same sequence as mice in the chronic stressor condition). On the 22^nd^ day, these mice were exposed to the social stressor just as mice in the acute stress condition had been, and then were decapitated 3 min afterward for collection of blood and brain.

#### Experiment 3: Influence of acute and chronic stressors on plus maze behavior

This experiment was conducted to assess the effects of the stressor conditions, precisely as described in Experiment 2, on anxiety-related behaviors measured on an elevated plus maze. The black Plexiglas plus-maze that was elevated 75 cm above floor level, had two arms (24.8 cm long×7.7 cm wide) enclosed by 21 cm high walls, while the remaining two identical arms were open. The maze was situated in a dimly lit room. As shown in Figure 1, 2 hr following exposure to the stressor, and then again 2 weeks later, mice again tested in the plus maze test. The procedure comprised mice being individually placed in one of the closed arms of the plus maze, facing away from the center area, and their behavior was recorded over a 5 min period by a ceiling-mounted video camera. The amount of time spent in each of the arms, the number of arm entries into the open and the closed arms (an arm entry defined as all four paws being placed in an arm of the plus-maze), were subsequently determined from the videotapes. Following testing of each mouse the plus-maze was thoroughly cleaned using 5% ethanol.

**Figure 2:**
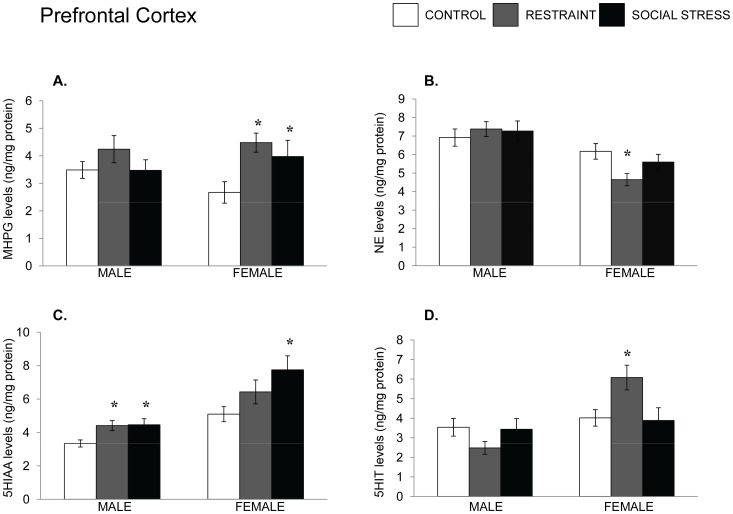
NE and 5-HT variations in the prefrontal cortex following an acute stressor. Mean (±SEM) concentration of MHPG and NE (Panels A and B) and 5-HIAA and 5-HT (Panels C and D) of the prefrontal cortex (PFC) among male and female mice that had experienced either no stress, restraint for 15 min, or a resident-intruder stressor. In males, the resident-intruder paradigm was essentially the same as that used in most social defeat paradigms (an intruder is placed in the cage of a resident who then defends his territory), whereas in females the paradigm comprised mice being placed in the cage of a lactating dam. *p<.05 relative to nonstressed mice of the same sex.

Ordinarily, when mice are first tested following a stressor experience, two antagonistic tendencies are initiated. One comprises increased fearfulness that promotes response inhibition especially in the context of the fear promoting stimulus, and the second comprises hyperarousal that is often seen as impulsivity-like behaviors (including rapid and frequent entries on to the open arms of a maze, entry into the center portion of an open field, or persistence in swimming in a forced swim test) [43]–[44]. However, when animals are retested several days later, when the initial arousal has abated, the behavior largely reflects fearfulness or anxiety, characterized by decreased active swimming (increased floating) in the forced swim test [43]. Thus, in the present investigation mice were tested 2 hr after the stressor and again 2 weeks later as the behavioral profiles at these times would be expected to be very different from one another.

### Corticosterone Determination

For corticosterone analyses, blood was collected in tubes containing 10 µg of EDTA, centrifuged for 8 min at 3600 RPM, and the plasma was stored at −80°C for subsequent corticosterone determination using a commercial radioimmunoassay RIA kit (ICN Biomedicals, CA). Corticosterone levels were determined, in duplicate, in a single run to avoid inter-assay variability, and the intra-assay variability was less than 8%.

### Monoamine Determinations

Levels of norepinephrine (NE) and serotonin (5-HT) and their respective metabolites, 3-metoxy-4-hydroxyphenylethyleneglycol (MHPG) and 5-hydroxy-3-indoleacetic acid (5-HIAA) in each brain region were determined using high-performance liquid chromatography (HPLC) as previously described [45]. Briefly, tissue punches were sonicated in a solution obtained from a stock solution containing 500 ml HPLC grade water, 5.0 ml methanol, 0.0186 g EDTA, and 14.17 g monochloroacetic acid. After centrifugation, 20 ml of the supernatant was passed at a flow rate of 1.5 ml/min (1400–1600 p.s.i.) through a system equipped with a M-600 pump (Milford, USA), a guard column, a radial compression column (5 m, C18 reverse phase, 8 mmr 10 cm), and a 3-cell coulometric electrochemical detector (ESA model 5100A). The mobile phase used for separation comprised 1.3 g heptane sulfonic acid, 0.1 g disodium EDTA, 6.5 ml triethylamine, and 35 ml acetonitrile that had been filtered using 0.22-mm filter paper, degassed, and the pH levels adjusted to 2.5 using phosphoric acid. A Hewlett-Packard integrator determined the height and area of the peaks. The protein content of each sample was measured using bicinchoninic acid with a protein analysis kit (Pierce Scientific, Canada), and a spectrophotometer (Brinkman, PC800 colorimeter). Amine and metabolite concentrations were based on protein levels. The lower limit of detection was 5.0 pg/ml.

### Data Analyses

As the social stressor used for males and females differed, the data for each of the outcome measures was analyzed independently for each sex. Plasma corticosterone levels, as well as levels of NE and 5-HT and their respective metabolites, MHPG and 5-HIAA in Experiment 1 and 2 were analyzed through a between groups one-way analysis of variance (ANOVA). Behaviors on the plus maze were analyzed by a mixed measures ANOVA with stressor as the between group variable and Time following treatment as the within group variable. Follow-up comparisons were performed using t tests with a Bonferroni correction to protect the α level at 0.05.

## Results

### Experiment 1: Restraint and Social Stressor Effects

The stressor treatments did not appreciably influence either MHPG or NE in the PFC of male mice, whereas among females the stressor treatment influenced the accumulation of MHPG and levels of NE, *F’s* (2,24) = 4.00 and 3.88, *p’*s <.05, respectively; see Figure 2A and B. The follow-up tests indicated that both restraint and intrusion into the cage of a lactating female increased MHPG levels to a comparable extent. As well, restraint significantly reduced NE, whereas the social stressor did not promote a significant effect in this regard. The accumulation of 5-HIAA was also increased by the stressor treatments in both males, F(2,24) = 4.44, p<.05 and in females, F(2.24) = 3.73, p<.05. The follow-up comparisons indicated that in males, both stressors increased the accumulation of 5-HIAA to a comparable extent, whereas in females the social stressor increased 5-HIAA relative to nonstressed mice (Figure 2C and D). Finally, in females the levels of 5-HT were elevated following exposure to the restraint stressor, F(2, 29) = 4.54, p<.05.

**Figure 3:**
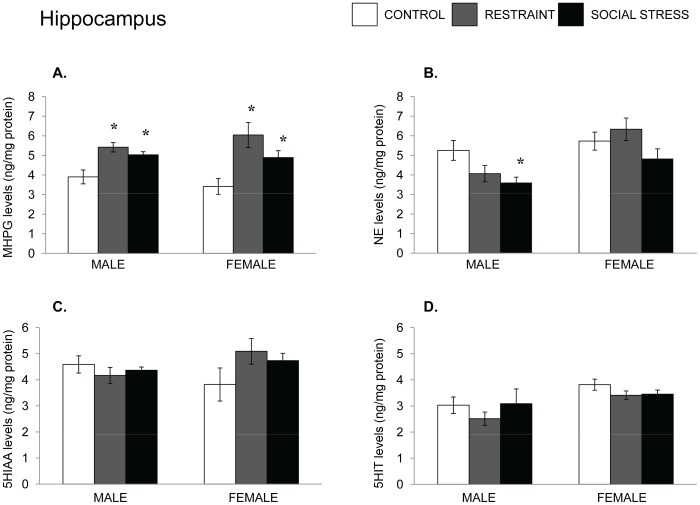
NE and 5-HT variations in the hippocampus following an acute stressor. Mean (± SEM) concentration of hippocampal MHPG and NE (Panels A and B) and 5-HIAA and 5-HT (Panels C and D) among male and female mice that had experienced either no stress, restraint for 15 min, or a resident-intruder stressor. *p<.05 relative to nonstressed mice of the same sex.

As depicted in Figure 3A and B, the Stressor condition influenced hippocampal MHPG accumulation among males and females, F(2,29) = 8.81, 5.91, p’s <.01 in that both stressors effectively increased the metabolite accumulation. Moreover, among the males the levels of NE declined, F(2,29) = 4.41, p<.05, primarily as a result of the effects of the social stressor, whereas in females the level of NE was not affected by the stressor. In contrast to the NE variations, hippocampal 5-HIAA and 5-HT were not affected by the stressor treatments in either the males or females.

**Figure 4:**
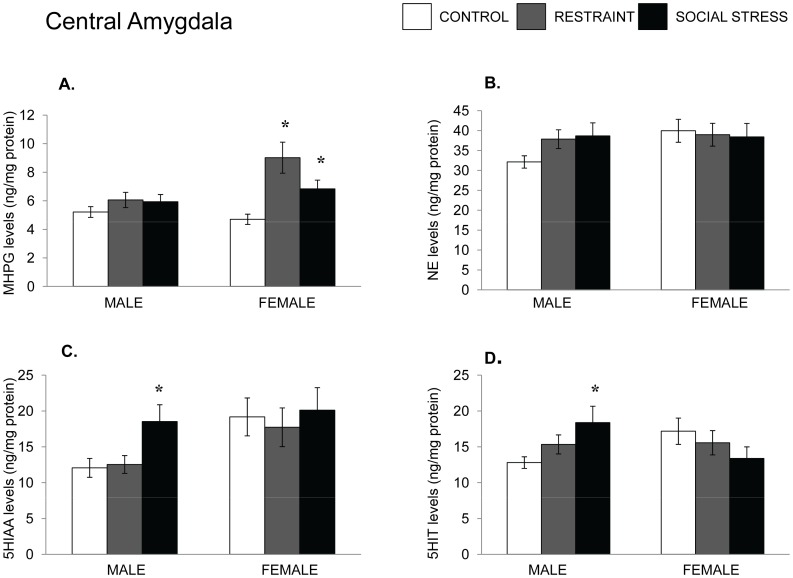
NE and 5-HT variations in the amygdala following an acute stressor. Mean (± SEM) concentration of MHPG and NE (Panels A and B) and 5-HIAA and 5-HT (Panels C and D) of the central amygdala among male and female mice that had experienced either no stress, restraint for 15 min, or a resident-intruder stressor. *p<.05 relative to nonstressed mice of the same sex.

Finally, analyses within the central amygdala indicated that MHPG accumulation was not significantly affected by the stressor in males, but both stressors increased MHPG accumulation in females, F(2,22) = 8.64, p<.01. The levels of NE were unaffected by the stressors in either sex (see Figure 3A and B). Finally, in the amygdala of male mice, the level of 5-HIAA was altered by the stressor, F(2,22) 3.22, p = .05, as was the level of 5-HT, F(2,22) = 3.96, p<.05. In both instances the social stressor increased levels, whereas restraint did not. In contrast to the 5-HT variations seen in males, neither 5-HIAA nor 5-HT within the amygdala was affected by the stressor in females (Figure 4C and D).

### Experiment 2: Effects of the Acute and Chronic Stressor Treatments on Plasma Corticosterone and Brain Monoamine Levels and Utilization

**Figure 5:**
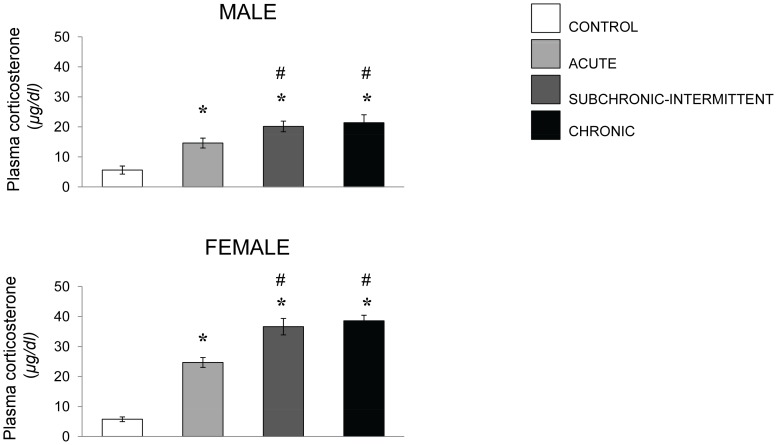
Corticosterone levels following an acute stressor. Mean (± SEM) plasma corticosterone concentrations (µg/dl) among male (upper) and female (lower) mice that had experienced either no stress, restraint for 15 min, or a resident-intruder stressor. *p<.05 relative to nonstressed mice of the same sex. # p<.05 relative to acutely stressed mice.

The concentrations of plasma corticosterone in males and in females were elevated in response to the stressor treatments, F (3,34 and 3,33) = 14.45 and 65.60, p’s <.001. In both sexes each of the stressors increased corticosterone levels, and the magnitude of the effects was greater after the chronic daily and chronic intermittent stressors than after the acute stressor treatments (Figure 5). However, corticosterone was measured at only a single time point following the stressor treatment, at a time that was likely well before the peak corticosterone effect emerged (3 min after stressor termination and 18 min after stressor introduction).

#### Brain monoamine levels and utilization

Table 1 provides an overview of the NE and 5-HT changes, and that of their metabolites, MHPG and 5-HIAA, respectively as a function of the stressor treatments. In general, the profile of amine changes in males differed from that evident in females, although as indicated earlier, direct comparisons between the sexes in response to the social stressor was inappropriate given that the stressors were somewhat different for the two sexes. Indeed, in contrast to males, among females there were no overt physical attacks against the intruder, and hence the stressor was solely of a psychogenic nature, largely comprising the threat of the lactating dam. It should be underscored, as well, that in Experiment 1 mice had been housed in groups of three, but as Experiment 2 entailed a chronic stressor procedure, mice were housed individually. Like others [46] we observed that isolated housing itself comprises a stressor [47], and hence the effects on the levels and utilization of monoamines in nonstressed mice, and those exposed to the acute stressor in Experiment 2, were not expected to be entirely congruent with those of Experiment 1.

**Table 1:** Summary of changes in MHPG, NE, 5-HIAA and 5-HT following acute, subchronic-intermittent and chronic stressors (Experiment 2).

		MALE			FEMALE		
		Acute Stressor	Subchronic-Intermittent Stressor	Chronic Stressor	Acute Stressor	Subchronic-Intermittent Stressor	Chronic Stressor
**PFC**	MHPG	**−**	**↑**	**↑**	**−**	**↑**	**−**
	NE	**−**	**−**	**−**	**↓**	**↓**	**↓**
							
	5HIAA	**↑**	**−**	**−**	**↑**	**↑**	**−**
	5-HT	**−**	**−**	**−**	**↓**	**−**	**−**
**Hippocampus**	MHPG	**−**	**↑**	**↑**	**−**	**↑**	**↑**
	NE	**−**	**−**	**−**	**−**	**−**	**−**
							
	5HIAA	**−**	**−**	**−**	**−**	**−**	**−**
	5-HT	**−**	**−**	**−**	**−**	**−**	**−**
**Amygdala**	MHPG	**−**	**↑**	**↑**	**↑**	**↑**	**↑↑**
	NE	**−**	**−**	**−**	**−**	**−**	**−**
							
	5HIAA	**−**	**↑**	**↑**	**−**	**−**	**−**
	5-HT	**↓**	**−**	**↓**	**−**	**−**	**−**

↑ relative to control group;

↑↑ relative to control, acute stressor and subchronic-intermittent stressor groups.

**Figure 6:**
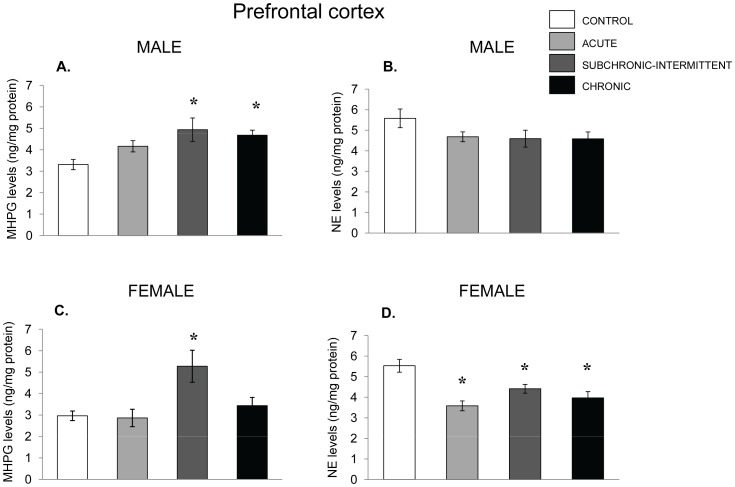
NE variations in the prefrontal cortex following chronic, sub-chronic and acute stressors. Mean (± SEM) concentration of MHPG and NE in the prefrontal cortex of male (Panels A and B) and female mice (Panel C and D) that had not been stressed (control), exposed to an acute stressor (resident intruder paradigms for the two sexes), a subchronic-intermittent stressor (7 stress sessions over 21 days) followed by the resident-intruder paradigm on Day 22, or a chronic stressor (variable stressor exposure on each off 21 days) followed by the resident-intruder stressor. *p<.05 relative to nonstressed mice.

Within the PFC of male mice, MHPG levels (Figure 6A) varied with the stressor treatment mice, F(3,34) = 11.37, p<.001. Exposure to an acute social stressor was not sufficient to increase NE utilization compared to nonstressed controls, although a 25% rise of the metabolite was apparent. However, the chronic treatments administered daily or intermittently, significantly increased NE utilization. This was accompanied by a small decline of NE levels in male mice (Figure 6B), which accounted for 10% of the variance, but did not reach an acceptable level of significance (p<.09).

In the PFC of female mice the utilization (Figure 6C) and levels of MHPG and NE (Figure 6D) differed as a function of the stressor condition, F’s (3, 30) = 6.11, 7.52, p<.005. The follow-up tests indicated that the accumulation of MHPG was increased solely among mice subjected to the subchronic-intermittent stress regimen, but the levels of NE were reduced in each of the stressor conditions relative to nonstressed mice. Thus, the effects of the acute stressor on MHPG and NE among females in Experiment 2 were less pronounced than in Experiment 1.

**Figure 7:**
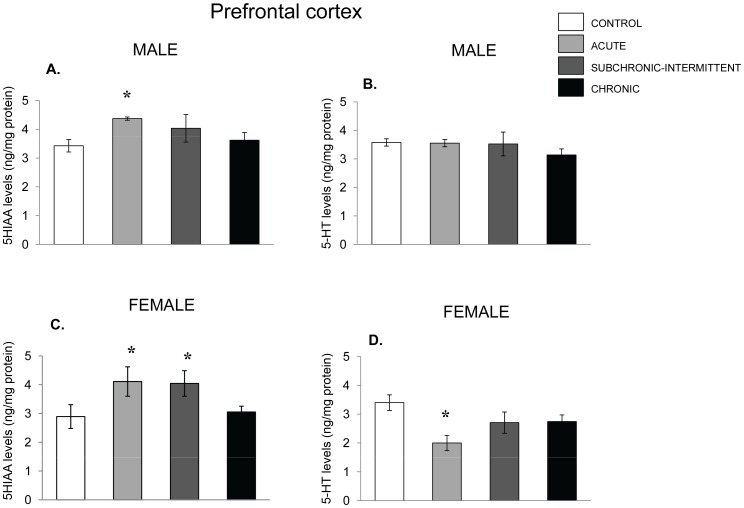
5-HT variations in the prefrontal cortex following chronic, subchronic-intermittent and acute stressors. Mean (± SEM) concentration of 5-HIAA and 5-HT in the prefrontal cortex of male (Panels A and B) and female mice (Panel C and D) that had not been stressed (control),exposed to an acute stressor (resident intruder paradigms for the two sexes), a subchronic-intermittent stressor (7 stress sessions over 21 days) followed by the resident-intruder paradigm on Day 22, or a chronic stressor (variable stressor exposure on each off 21 days) followed by the resident-intruder stressor. *p<.05 relative to nonstressed mice.

Although 5-HIAA accumulation within the PFC of male mice was affected by the stressor, F(3,30) = 3.82, p = .02 (see Figure 7A and 7B), the levels of 5-HT did not vary with the stressor treatment.,. The follow-up tests indicated that among males the acute stressor increased the levels of the metabolite, whereas in the chronically stressed mice this outcome was not statistically significant. Among female mice, group differences were also apparent, F (3,30) = 3.27, p = .03, and were attributable to a rise of the metabolite in the acute stress and the subchronic-intermittent stressor conditions. The 5-HT levels in the PFC of female mice varied as a function of the treatment administered, F’s (3, 30) = 3.53, p<.05, which was attributable to reduced 5-HT levels among female mice that had been exposed to the acute social stressor, whereas the levels of the parent amine were not significantly reduced following the chronic stressor treatments (Figure 7D).

**Figure 8:**
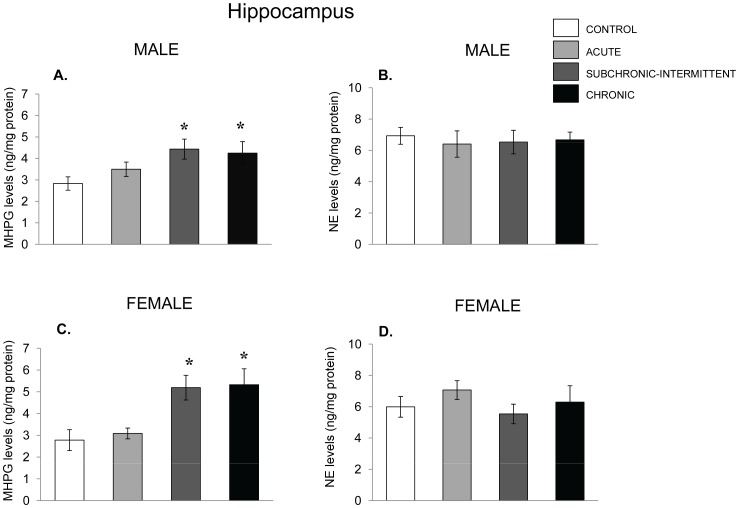
NE variations in the hippocampus following chronic, subchronic-intermittent and acute stressors. Mean (± SEM) concentration of MHPG and NE in the hippocampus of male (Panels A and B) and female mice (Panel C and D) that had not been stressed (control), exposed to an acute stressor (resident intruder paradigms for the two sexes), a subchronic-intermittent stressor followed by the resident-intruder paradigm, or a chronic stressor followed by the resident-intruder stressor. *p<.05 relative to nonstressed mice.

Hippocampal NE and MHPG variations were very similar in males and females. In males the accumulation of MHPG (Figure 8A) varied as a function of the stressor treatment the mice received, F(3,32) = 3.31, p<.05, being elevated in both the subchronic-intermittent and chronic stressor conditions, but not in response to the acute stressor. The NE levels (Figure 8B) did not vary with the stressor treatment. It likewise appeared that in females, hippocampal MHPG (Figure 8B) was increased by the stressor, F(3,30) = 6.12, p<.01, reflecting increased metabolite accumulation following the subchronic-intermittent and chronic conditions, but not by the acute stressor (Figure 8D). The levels of NE, in contrast, were not affected by any of the treatments. Moreover, the stressors did not influence either 5-HT or 5-HIAA in either males or females (data not shown).

**Figure 9:**
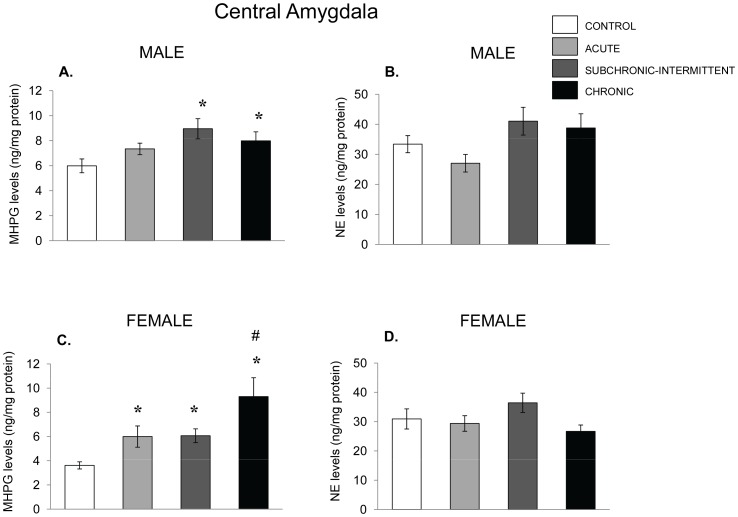
NE variations in the central amygdala following chronic, subchronic-intermittent and acute stressors. Mean (± SEM) concentration of MHPG and NE in the central amygdala of male (Panels A and B) and female mice (Panel C and D) that had not been stressed (control), exposed to an acute stressor (resident intruder paradigms for the two sexes), a subchronic-intermittent stressor followed by the resident-intruder paradigm, or a chronic stressor followed by the resident-intruder stressor. *p<.05 relative to nonstressed mice of the same sex. # p<.05 relative to acutely stressed mice.

The levels of NE within the amygdala of male mice (Figure 9C) were not affected by any of the treatments, whereas NE utilization (Figure 8A) varied as a function of the stressor treatment the mice received, F(3,32) = 3.31, p<.05. Once again, the follow up test showed that NE utilization increased among mice that experienced the subchronic-intermittent or chronic stressor treatments. In the amygdala of female mice, NE levels were also unaffected by the stressors (Figure 9D), whereas the utilization of NE varied as a function of the stressor treatment mice received. F(3,33) = 5.89, p = .002. The follow-up tests indicated that relative to nonstressed controls all the stressor conditions were effective in increasing NE utilization. This increased utilization was most pronounced following chronic stressor as MHPG in this condition exceeded that evident in the other stressed groups.

**Figure 10:**
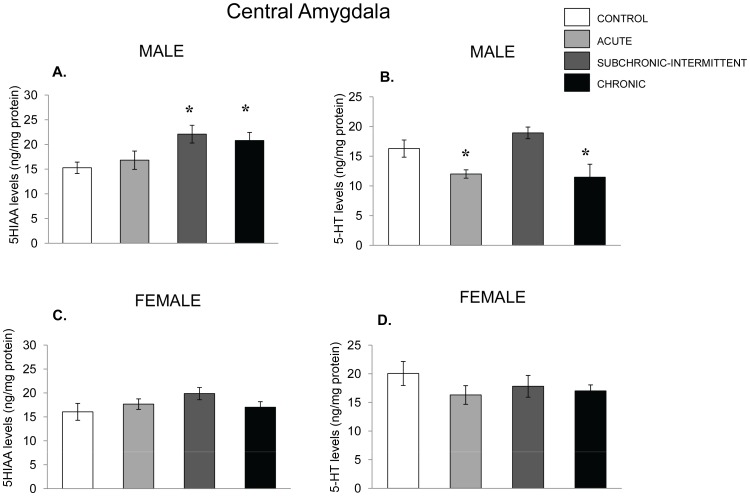
5-HT variations in the central amygdala following chronic, subchronic-intermittent and acute stressors. Mean (± SEM) concentration of 5-HIAA and 5-HT in the central amygdala of male (Panels A and B) and female mice (Panel C and D) that had not been stressed (control), exposed to an acute stressor (resident intruder paradigms for the two sexes), a subchronic-intermittent stressor followed by the resident-intruder paradigm, or a chronic stressor followed by the resident-intruder stressor. *p<.05 relative to nonstressed mice of the same sex.

The 5-HT and 5-HIAA in the amygdala of male mice varied as a function of the stressor treatment, F(3,31) = 7.14, 3.86, p ‘s <.001 and.01, respectively. Follow up test showed that compared with nonstressed controls both the subchronic-intermittent and chronic treatments significantly increased 5-HIAA accumulation (Figure 10A), whereas 5-HT levels (Figure 10B) were reduced by exposure to acute and chronic stressed mice, but not by the subchronic-intermittent stressor regimen. In contrast to the effects seen in males, among females the stressor treatments provoked only a modest, nonsignificant, rise of 5-HIAA and a small nonsignificant decline of 5-HT.

### Experiment 3: Effects of the Stressor Treatments on Anxiety Measured in a Plus-maze Test, in Males and Females Mice

**Figure 11:**
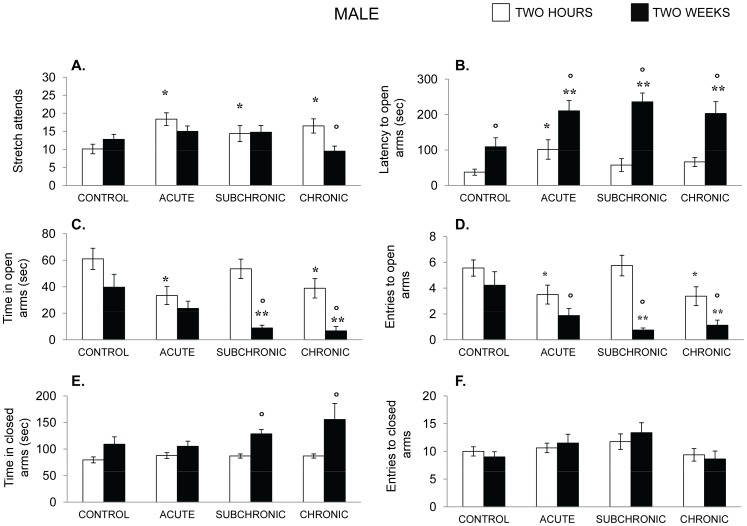
Effects of chronic, subchronic-intermittent and acute stressors on anxiety-related behaviors in male mice. Mean (± SEM) stretch attend responses (A), latency to enter the open arms (B), Time in the open Arms (C), Number of entries into the open arms (D), Time in the closed arms (E) and number of entries into the closed arms 2 he and again 2 weeks following treatment. Male mice had either not been stressed (control), exposed to an acute stressor (resident intruder paradigms for the two sexes), a subchronic-intermittent stressor followed by the resident-intruder paradigm, or a chronic stressor followed by the resident-intruder stressor. *p<.05 relative to nonstressed mice. ^o^ p<.05 relative to performance measured 2 hr following the treatment.

The plus maze performance of male mice is shown in Figure 11. In the males, the number of stretch attend responses emitted varied with the Stressor treatment×Time of testing interaction, F(3, 29) = 4.76, p<01. The follow up tests indicated that when stressed mice were tested 2 hr after the stressor treatment they made more stretch-attend responses than did nonstressed animals. However, upon retesting 2 weeks later, this effect was absent, and indeed, those mice that that had been exposed to the chronic stressor on each of 21 days emitted fewer stretch-attend responses than did nonstressed mice.

The latencies to enter the open arms varied as a function of the Stress treatment×Time interaction, F (3,29) 2.88, p = .05. The follow up tests revealed that when mice were tested 2 hr after stressor exposure the latency to enter the open arm was longer in acutely stressed mice than in controls, but this effect was not evident among chronically stressed mice, seemingly suggesting a degree of adaptation to the stressor. However, upon retesting mice 2 weeks later the latencies to enter the open arms of the maze was longer in each of the groups, but particularly so in those mice that had been exposed to a stressor treatment.

The analyses also revealed that the number of the entries into the open arms, as well as the time spent on the open arms (Figure 11C and 11D), varied as a function of the Stressor condition×Time following the initial stressor experience interaction, F’s(3, 29) = 4.90, 5.67 p’s <.01, respectively. The follow up comparisons of the simple effects comprising this interaction indicated that both the acute and chronic unpredictable stressor reduced the number of open arm entries and mice in these conditions spent less time on the open arms relative to nonstressed mice; curiously, this outcome was not evident in mice that had been exposed to the subchronic-intermittent stressor regimen. When animals were retested 2 weeks later, the entries into the open arms were reduced in each of the stressed groups. In fact, the chronically stressed mice performed, on average, only a single open arm entry and spent less than 10 sec on these arms, and thus a floor effect might have limited more pronounced effects from being detected.

In contrast to responses to the open arms, the stressor treatment did not influence the number of closed arm entries, although the time spent on the closed arms varied as a function of the test session, F(3, 29) = 24.45, p<.01. Essentially, the time spent in the closed arms increased over the two sessions irrespective of the condition. This effect was somewhat greater in the chronically stressed mice, but this was largely related to appreciably greater variance, and the extent of the rise was not greater than that evident in the remaining groups.

**Figure 12:**
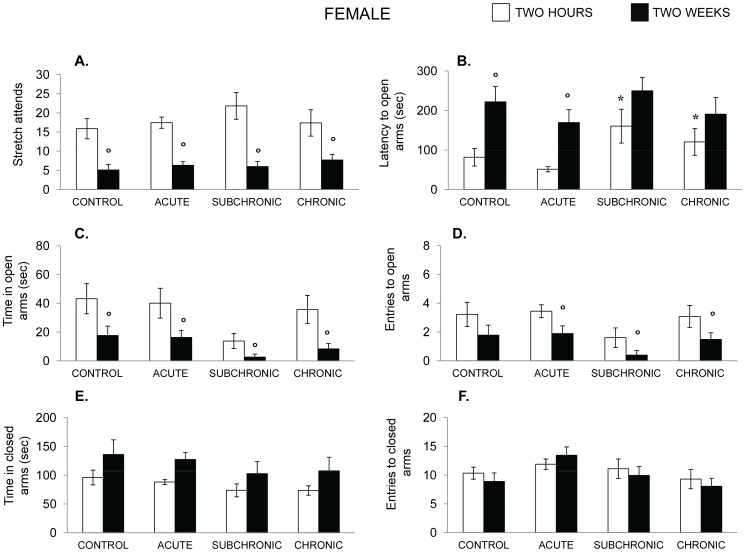
Effects of chronic, subchronic-intermittent and acute stressors on anxiety-related behaviors in female mice. Mean (± SEM) stretch attend responses (A), latency to enter the open arms (B), Time in the open Arms (C), Number of entries into the open arms (D), Time in the closed arms (E) and number of entries into the closed arms 2 he and again 2 weeks following treatment. Female mice had either not been stressed (control), exposed to an acute stressor (resident intruder paradigms for the two sexes), a sub subchronic-intermittent chronic stressor followed by the resident-intruder paradigm, or a chronic stressor followed by the resident-intruder stressor. *p<.05 relative to nonstressed mice. ^o^ p<.05 relative to performance measured 2 hr following the treatment.

The performance of female mice, depicted in Figure 12, could be distinguished from that of males in several respects. Stretch attend responses in females were only modestly elevated by the stressor treatments, F(3,30) = 2.30, p = .09, but these responses, as in males, markedly declined over the two sessions, F(1, 30) = 74.094, p<.001 (Figure 11A). Furthermore, the latency to enter the open arms varied as a function of the stressor treatment, F(3,30) = 4.86, p<.01 as well as the test session, F(1,30) = 21.16, p<.01. The follow up tests indicated that on the first test session the mice in the two chronic stressor conditions took longer to enter the open arms than did those mice that had not been stressed or mice that had been exposed to the acute stressor (Figure 12B). On the second test 2 weeks later, the latencies to enter the open arms were longer than on the first test in all of the groups, but did not vary significantly with the condition, and as seen in Figure 12B, the effects of the subchronic-intermittent and chronic treatments were no longer evident relative to mice in the other groups.

The number entries onto the open arms as well as the time spent on these arms among the females were relatively variable. The analyses indicated that the frequency of entries and the time spent on the open arms declined over the two sessions, F’s (1,30) = 16.34, 23.82, p’s <.01 (Figure 12C and 12D). The stressor treatment did not affect either the number of such entries or the time on the open arms, although the time in the open arms among mice that had been exposed to chronic stressors was just shy of significance, F(3,30) = 2.71, p = .06. Once again, follow-up tests were conducted on the basis of the a priori hypotheses that had been made concerning the Treatment×Time interaction. These tests indicated that the subchronic-intermittent treatment resulted in a decline of the entries and the time spent on the open arms relative to nonstressed mice. This was especially notable when mice were tested 2 weeks after the stressor treatments. As depicted in Figure 12C and 12D, the entries to these arms and the time spent in these arms were exceptionally brief, and as in males, a floor effect might have precluded more prominent differences from being detected. Unlike these varied behavioral changes, the stressors did not influence time in the closed arms or the number of entries into the closed arms, although there was a modest increase of closed arm entries over the two sessions.

## Discussion

Analyses of agonistic behaviors using the resident-intruder paradigm has been investigated for more than 3 decades [48], but has enjoyed a rejuvenation with the demonstration that this seemingly ethologically-relevant stressor might be useful to model depression and anxiety [20], [49], [50] or specific features of depression [51] and stressor-provoked drug self-administration [52], [53]. However, as indicated earlier, with only a few exceptions (e.g., [52], [54], this paradigm has primarily been used with male animals, despite the fact that stress-related pathologies are more common in females than in males. Although males are aggressive toward another male interloper in their territory, they are less likely to exhibit threat behaviors towards females. Thus, an alternative paradigm was used in which females intruded upon the territory of a lactating female, which ordinarily elicits aggressive gestures from the resident mouse [26]–[28], which provokes several biological changes akin to those ordinarily associated with the resident-intruder paradigm in males [31]. In the present investigation it is shown that although the resident intruder paradigms in males and females involved different procedures (and in females the procedure did not elicit any occasions of physical assault), thus making direct comparisons between the sexes inappropriate, it is clear that the procedure used in females elicited corticosterone and brain monoamine changes that were at least as strong (depending on brain region) as those provoked by a stressor (restraint) that was common to both males and females.

In males that had been acutely exposed to the resident-intruder paradigm, in the absence of earlier stressors (i.e., in the acutely stressed mice), a moderate anxiety-like response was provoked that comprised elevated reluctance to enter the open arms of the plus-maze and diminished time spent on the open arms. This effect was markedly greater when mice were retested 2 weeks afterward. Similarly, although the chronic stressor regimen did not elicit a change of behavior measured 2 hours after the stressor terminated, anxiety-like behavior was apparent upon retesting 2 weeks later, and this effect was stronger than it was among mice that had been exposed to the acute stressor. It might have been thought that the limited anxiety evident when mice were tested 2 hr after the chronic stressor might have reflected an adaptation, but the fact that the anxiety was as profound as it was 2 weeks later is obviously inconsistent with this view. As indicated earlier, the period following a stressor is often accompanied by hyperarousal and impulsivity, so that when animals are tested in certain situations, including the plus-maze, the hyperarousal/impulsivity obfuscates the anxiety or the depressive-like state that might concomitantly be present [31], [43], [44]. By allowing for a decline of arousal/impulsivity with the passage of time, anxiety stemming from the original stressor and its association with the plus-maze can emerge, uncontaminated by other effects stemming from the stressor.

The finding that the stressor would have pronounced anxiogenic effects two weeks later was not unexpected, but it should be considered that the appearance of this persistent outcome might have been tied to the specific context in which mice had been tested. By example, it was reported previously that acute stressors can have persistent consequences, but this depended on the test procedure that was employed [55]. If mice had been exposed to an uncontrollable stressor they exhibited a marked disruption of responding for rewarding brain stimulation that was assessed immediately afterward and then again 1 and 7 days later. Indeed, the disrupted responding appeared to become more pronounced at the longer interval following the stressor session. Importantly, this protracted outcome was only evident if animals were initially tested soon after the stressor session. If animals were exposed to an uncontrollable stressor and then tested for the first time one week later, then there was no evidence of behavioral disturbance. Essentially, the uncontrollable stressor had to be paired with the test situation for any persistent effects to be evident. In the present investigation mice likewise exhibited marked anxiety-like behaviors two weeks after the social stressor, long after any anxiogenic effects of the stressor would ordinarily be expected. In this instance, mice had initially been tested 2 hr after the social stressor and it is possible that this initial test might have been necessary for the protracted effect of the stressor to become apparent. In effect, the treatment might not have elicited a generalized anxiety, but rather one that was tied to cues that had been associated, at least to some extent, with initial stressor experience.

As indicated earlier, it has frequently been observed that the behavioral and neurochemical profiles elicited by stressors were very different in males and females that had been exposed to a stressor [5], [6], [9], [56]. In the present investigation, the profile of behavioral changes induced by the stressor in females was reminiscent of that seen in males, although it was clearly less pronounced when mice were tested 2 hr after the social challenge. On the one side, the limited behavioral changes might be attributable to females being more resilient to the effects of stressors [5], [6], [9], [56]–[59]. On the other, it is possible that the stressor to which females were exposed was not a very powerful one, and indeed, in this paradigm females did not exhibit explicit aggressive behaviors, such as attacks and biting as often seen in males. Yet, it was clear that the intruder paradigm in females was effective in eliciting distress, reflected for instance by the increased anxiety associated with the chronic treatments, as well as by the elevated corticosterone levels and monoamine variations that were observed in females. Indeed, as reported using other stressors, the corticosterone rise in the present investigation appeared to be appreciably (>50%) greater in females than in males.

Monoamine variations have been implicated in anxiety disorders as well as depression, and aggressive behaviors have been associated with variations of 5-HT [60]. Moreover, in rats, 5-HT levels in the PFC are generally higher in females than in males [57], but sexual dimorphisms regarding stressor effects, particularly in relation to conspecific aggression, have been less well studied. Thus, it was of particular interest to determine the effects of a resident-intruder stressor on brain region-specific variations of NE and 5-HT activity relative to a stressor that is commonly used in both males and females (restraint), and to determine whether these effects might vary in the context of a previous chronic stressor experience**.** In this regard, it has frequently been reported that the increased NE and 5-HT utilization and the reduced levels of these amines that accompany acute stressors are not apparent following chronic homotypic stressors [1]. However, these monoamine disturbances are more likely to persist if animals are exposed to a chronic, variable, unpredictable stressor regimen [1] and may also be accompanied by altered 5-HT_1A_, 5-HT_2A_ and 5-HT_2c_ receptor expression, although these changes varied with the brain region examined [61], [62]. Thus, in the present investigation we also assessed whether the effects of the social stressor would be altered when superimposed on a backdrop of a chronic or subchronic-intermittent stressor regimen.

Consistent with the effects of other stressors [1], in the present investigation both restraint and the presence of an intruder increased the accumulation of MHPG in the prefrontal cortex, hippocampus and amygdala of females, but this outcome in males was limited to the hippocampus. Unlike this preferential stressor effect in females, 5-HIAA accumulation was elicited by the social stressor in the PFC of both males and females, and was more likely to occur in the amygdala of male mice, although it has also been reported that 5-HT variations in rats may be greater in females [62]. Given the procedural differences as well as the differences in species, it is uncertain what might have contributed to the different effects seen within the amygdala of stressed animals. It will be recalled that in response to strong stressors amine levels may be reduced when utilization exceeds its synthesis [1]. This outcome was infrequently observed in the present investigation, and when this did occur, it was not more prevalent in one sex over the other. For instance, in the PFC, the NE reductions elicited by the stressor were more common in females, whereas in the hippocampus the NE reductions were more apt to be evident in males. In the central amygdala, the utilization of NE was also elevated by the stressor conditions in both males and females, and became more pronounced with chronic stressors than with the acute treatment.

As previously reported [63]–[64], in mice that had been exposed to a chronic or subchronic-intermittent stressor comprising a series of different insults, the utilization of NE and 5-HT tended to be greater than that evident with acute stressor exposure, although with the chronic stressor the effects on 5-HIAA within the PFC were less notable than after an acute stressor. In other regions (e.g., amygdala and hippocampus) the extent of the NE utilization elicited, reflected by elevated MHPG accumulation, was increased by the chronic stressor to a greater extent than it was by the acute stressor treatment, and once again this was apparent in both males and females. The changes of 5-HIAA were also more likely to be elevated in the amygdala of male mice (but not females), an outcome that has also been seen following acute restraint [62]. In contrast, in the PFC the utilization of 5-HIAA, which was elevated by an acute stressor, was not similarly increased with the chronic stressor in either males or females. Furthermore, the diminished 5-HT levels that accompanied the increased utilization elicited by the acute stressor in females, was not apparent following the chronic stressor. Ordinarily, it would be supposed that the regulation of 5-HT levels would be due to a compensatory increase of synthesis to meet the elevated utilization. Yet, as 5-HIAA accumulation dropped off with the chronic stressor, it is possible that moderation of utilization, rather than a compensatory increase of synthesis were responsible for the normalization of the 5-HT level.

It generally seemed that the effects on monoamine utilization and levels were more pronounced after chronic than after acute stressors, but these effects were specific to certain brain regions. There have been diverse reports concerning the effects of chronic unpredictable stressors on monoamine activity, but the factors that contributed to these outcomes are not readily identifiable as the procedures that have been used across studies have varied. In the case of intermittent chronic stressors, some studies have used mild stressors like those described by Willner [35], whereas others have used somewhat more intense stressors. Moreover, some studies were conducted in rats, whereas others involved different strains of mice, and as far as we know, none examined a social stressor as the last in a series of chronic challenges, making it that much more difficult to compare the monoamine variations across studies.

The present findings bring to mind several fundamental issues. First, although it is inappropriate to make direct comparisons between males and females given that the social stressor was different for the two sexes, it is significant that while the females showed greater NE variations in the PFC than did the males, the 5-HT variations in the amygdala occurred preferentially in the males. These data suggest that males might be more or less resilient than females with respect to certain neurotransmitters and in particular brain regions, whereas females may be more resilient with respect to particular neurotransmitter changes in still other brain regions. Moreover, these differences might play out with respect to particular pathologies. Thus, for instance, some neurochemical changes associated with chronic stressors (e.g., NE variations in the amygdala and PFC) might contribute to anxiety or PTSD vulnerability, whereas variations, such as 5-HT changes within the PFC might, potentially, be more closely aligned with depressive disorders. Of course, the present study was limited to monoamine levels and utilization, and further changes occur with respect to both NE and 5-HT receptor variations (e.g., [65]–[68], and it is certainly possible that the effects of allostatic overload might be evident with these receptor changes or further downstream processes. Moreover, resilience (or vulnerability) might reflect the conjoint or interactive consequences of several systems, including growth factors such as BDNF and FGF-2, and it is likely overly simplistic to assess allostatic overload pathology in the context of single biological systems.

Summarizing, it seems that the resident-intruder paradigm (social defeat) in males is effective in promoting a variety of neurochemical changes that have been implicated in psychological disorders, such as anxiety and depression, and that the nature of these effects is influenced by the animal’s preceding chronic or subchronic-intermittent stressor experiences. It likewise appeared that when a female intrudes into the cage of a lactating conspecific neurochemical changes are elicited in the intruder that are reminiscent of those seen in males, and in several respects these outcomes are more pronounced. Whether these neurochemical changes reflect greater vulnerability to stressors, increased biological changes that favor effective coping, or precursors to allostatic overload is uncertain. As already indicated, it has been suggested that the social defeat paradigm (and by extension the intruder paradigm in females) might be used to model anxiety and depression. It has, however, been suggested that social defeat, even when applied on a chronic basis, might not be a ‘pure’ model of depression, but might be more appropriately used to simulate anxiety and especially social avoidance [53]. Others have also used this paradigm to model PTSD [69], [70]. In fact, previous studies have shown that social defeat and inescapable tail shock, which are typically used to promote depressive-like behaviors in animal models, elicit several common neurochemical effects, but they also promote some important differences with respect to the neurochemical and hormonal changes elicited [50]. Thus, different stressors might engage several common neural circuits as well as some that are unique to particular stressors [71].
